# Monitoring of Communication Precursors in Extremely Low Birth Weight (ELBW) Newborns by Video Analysis Method: Preliminary Results

**DOI:** 10.3390/children9050602

**Published:** 2022-04-23

**Authors:** Laura Sundas, Silvia Palma, Marisa Pugliese, Maria Federica Roversi, Enrico Apa, Alberto Berardi, Elisabetta Genovese, Daniele Monzani

**Affiliations:** 1 Child and Adolescent Mental Health Service AUSL, 41121 Modena, Italy; l.sundas@ausl.mo.it; 2 Audiology, Primary Care Dep. AUSL, 41100 Modena, Italy; 3 Neonatal Intensive Care Unit, Department of Medical and Surgical Sciences for Mothers, Children and Adults, University of Modena and Reggio Emilia, 41100 Modena, Italy; marisa.pugliese@unimore.it (M.P.); roversi.federica@aou.mo.it (M.F.R.); alberto.berardi@unimore.it (A.B.); 4 Audiology, ENT Dept., University of Modena and Reggio Emilia, Via del Pozzo 71, 41124 Modena, Italy; enrico.apa@unimore.it (E.A.); elisabetta.genovese@unimore.it (E.G.); daniele.monzani@unimore.it (D.M.)

**Keywords:** communication precursors, GMDS-R, extremely low birth weight, preterm birth, video analysis

## Abstract

Background: The survival of extremely low birth weight infants (ELBW) has increased worldwide. Even in the absence of major disabilities, ELBW infants show difficulty in simple language functions. It is relevant to assess early abilities, which are the base of early linguistic skills, in order to implement customized intervention programs in ELBW infants. Aims: To evaluate communication precursors of language development in ELBW infants at 12 and 24 months of correct age (C.A). To investigate the correlation of linguistic and communicative prerequisites with mental development outcome at 24 months CA. Method: 52 ELBW neonates (mean gestational age 26.6 weeks, mean birth weight was 775 g) who were admitted to the neonatal intensive care unit of the University Hospital of Modena, were enrolled. Data were collected from archived audio-video recordings of neurodevelopmental follow-up visits. Video analysis of communicative and linguistic developmental was performed at 12 and 24 months CA. Neurodevelopmental outcome was evaluated with Mental Developmental Scales (GMDS-R). Results: The video-analysis showed that infants at 12 months CA used predominantly eye contacts and gestural turns, while vocal turns were scant. At 24 months CA, a significant change in eye contacts, vocal turns, gestural turns, and utterances (*p* < 0.001) occurred. The total number of utterances (*p* = 0.036) and eye contacts (*p* = 0.045) were significantly correlated to the Development Quotient (DQ) of Hearing and Language scale. Moreover, a significant correlation was found with the Personal-Social scale vocal turns (*p* = 0.009) and the total number of utterances (*p* = 0,02). Finally, the Global Quotient of the GMDS-R was related to the Vocal Turns (*p* = 0.034) and the total number of Utterances (*p* = 0.013). Conclusions: ELBW infants at 12 months CA use predominantly eye contacts and gestural turns to communicate with adults. At 24 months CA, the child’s communicative intention evolves from gestural to verbal communication. The latter is characterized by an increase in both vocal turns and the number of utterances produced during interaction. The video analysis we implement appears to be a sensitive tool for early assessment of communication and language development and to refine early intervention

## 1. Introduction

The survival of preterm newborns has increased worldwide, and premature birth (before 37 weeks of pregnancy) is the most common perinatal risk [1]. Preterm infants frequently suffer from delay of neuromotor and language development, and the severity of problems increases with decreasing gestational age [2]. For instance, very preterm newborns show a slower acquisition in word comprehension and production with increasing divergence compared to full-term peers from the age of 12 to 24 months [3,4,5]. Even in the absence of major disabilities, very preterm babies show difficulty in simple language functions [6].

Human communication skills develop early, and infants understand words before they can speak [7]. Contextually, the development of the auditory system is determinant. It has been shown that between the 23rd and 25th weeks of pregnancy, the cochlea is already structured, and between the 26th and 30th weeks, the fetus is able to detect and react to sound stimuli [8]. A very short time after postnatal exposure, infants [9] are able to discriminate between different prosodic patterns [10] and turn-taking is possible through a variety of non-linguistic cues as early as in the second month of life, long before any access to lexical information [11].

The first step of language development is characterized by the presence of prelinguistic skills, such as babbling, pointing, and making eye contact, the so-called “prelingual phase” (from birth to about 12 months of age) [12]. Subsequently, in the early-lingual phase (from 1 to 2.6 years of age), children show signs of word comprehension and start producing isolated words and short sentences. Finally, from 2.6 to 5 years of age, grammar starts to develop and sentences become more complete [13]. It is relevant to assess early abilities, which are the base of early linguistic skills, in order to implement customized intervention programs.

Documenting advances in language development requires a reliable staging system that can be applied across the continuum of vocalization types produced during the first 2 years of life [14]. This task is complicated by the adoption of different methods for assessing varying aspects prelinguistic skills, making comparison of studies difficult, also in observations of full-term babies [15].

To this aim, a video recording technique was developed to observe child–adult interactions in a controlled setting and analyze various aspects of the interaction in deaf children [16], in particular after hearing rehabilitation. The technique was proven to be an objective and sensitive measure of the progresses through the preverbal and early verbal stages of language development [17,18], and, more recently, it has also been used to test the efficacy of intervention in deaf children with complex developmental needs [19].

To assess language development by a staging system suitable for daily clinical practice, early communications of a cohort of extremely low birth weight infants (ELBW) were investigated through video analysis. The methodology was based on the experiences [17,18,20] about monitoring of language development for children with hearing impairment. An Italian adaptation [21] of transcriptive method of communication precursors was used during child–adult interactions in follow-up protocol visits for ELBW.

The first aim of this study was to evaluate communication precursors of language development in ELBW infants at 12 and 24 months of correct age (CA) by the mean of the above-mentioned video-analysis technique. A second purpose was to investigate the correlation of linguistic and communication precursors with mental development outcome at 24 months CA assessed by the GMDS-R [22].

## 2. Materials and Methods

Observational retrospective study. ELBW infants admitted to the Neonatal Intensive Care Unit of Azienda Ospedaliero-Universitaria of Modena underwent a follow-up protocol regarding neuromotor and mental development up to 24 months CA. The follow up protocol includes: a standard neurologic examination, an audiological evaluation and an assessment of child mental development by the use of the GMDS-R (0-2 years) [22]. The follow-up visits are usually video recorded and stored. Informed consent was requested from parents.

Inclusion criteria: neonates with a birth weight under 1000 g (extremely low birth weight neonates, ELBW). They were enrolled as the video recording of follow up visits was available. All the neonates had normal auditory brainstem response (ABR) and Otoacoustic Emissions with “clear” response.

Exclusion criteria: congenital malformations, genetic syndromes, major brain damage, visual impairment, hearing impairment.

### 2.1. Griffiths Mental Development Scale (GMDS-R)

The GMDS-R (0–2 years) provides a Global Development Quotient (DQ) of infants’ abilities with a mean of 100 and a standard deviation (SD) of 15 and five subscale quotients (Locomotor, Personal-Social, Hearing and Language, Eye and Hand Coordination, and Performance). The cut-off for abnormality is 2 SD. Locomotor measures the development of basic motor skills such as posture, walking, running and climbing. The Personal-Social Scale measures adaptive and self-help behavior at home and social attitude as evident in child–adult interaction. The Hearing and Language Scale evaluates the development of hearing by measuring responses to external sounds and speech and the development of language by measuring the production of sounds and words. The Eye and Hand Co-ordination Scale evaluates manipulative skills that requires fine motor handwork and visual acuity. The Performance Scale examines manipulative motor skills by assessing speed and precision at work. The assessments at the age of 24 months are reported.

### 2.2. Video Recording Session

The video recording of each follow-up visit was re-analyzed for approximately 15 min. A developmental speech therapist made a transcription and quantification of the communication precursors of children, at 12 and 24 months CA. Antecedent of communication expresses how the child responds to adult solicitation in the interaction. The assessment grid is based on the temporal organization of adult–infant turn-taking sequences, which is similar to that of adult verbal conversation. Observation included at least 10 interaction attempts on behalf of the adult. During each follow-up visit the speech therapist transcribed the amount and the type of communication precursors expressed by children. In case of doubt, video recordings have been viewed more times.

“Eye contact” occurs each time the infant directs its attention towards the practitioner by looking in his eyes; it corresponds to a 2 s sustained eye contact.

“Vocal turn taking with eye contact” indicates an infant’s gestural intention.

“Vocal turn taking without eye contact” expresses the infant’s communicative intention. “Gestural turn” taking indicates the infant’s feedback to the adult’s interaction proposal. “Communication initiative”, both gestural and vocal represents the infant’s autonomous communication initiative expressed in three ways: vocal, gestural, and cross-modal.

In contrast to evaluation in deaf children, which was conducted in thematic play settings, setting for ELBW children took place during a routinary clinical follow up.

The room had adequate lighting; the child’s face was framed by the camera monitoring the direction of the child’s gaze. During the session, adults adapted to the communication level of the child; neither examiner nor parent facilitated actions towards the child.

### 2.3. Statistical Analysis

One sample the Kolmogorov–Smirnov test was used to test if variables of the video recording protocol followed a normal distribution.

A paired sample *t*-test was the procedure adopted to determine whether the mean difference between two sets of observations was zero (12 and 24 months CA analyses) and Pearson correlation coefficient (r) was computed as a measure of the strength of a linear association between two variables (Video recordings variables, GMDS-R subscales). Statistical significance level was *p* < 0.05 in all procedures.

## 3. Results

Audio-video recordings of 52 ELBW newborns were collected. Seven neonates were excluded due to visual impairment (*n* = 2), hydrocephalus (*n* = 1), genetic syndrome (*n* = 1) and missing data (*n* = 3). Therefore, the final analysis was performed on the remaining 45 infants. There were 26 males and 19 females, the mean gestational age was 26.6 weeks, and mean birth weight was 775 g.

As neonates were evaluated between 2000 and 2004, more detailed demographic characteristics (maternal education level, etc.) could not be retrieved.

The preliminary Kolmogorov–Smirnov test documented *p* values between 0.087 and 0.912 so that the null hypothesis that variables were not normally distributed was rejected.

A transcription and quantification of verbal communication precursors was based on the video-analysis. Table 1 shows communication precursors at 12 and 24 months of CA. At 12 months CA, infants demonstrated to use predominantly eye contacts and gestural turns while vocal turns were scant. Table 2 shows video analysis of communication precursors at 12 and 24 months CA. There were significant changes in all variables. Interestingly, vocal turns and the number of autonomous communicative initiatives increased significantly from 12 to 24 months CA; furthermore, eye contacts and gestural turns to communicate with adults decreased.

Paired sample *t*-test between the two observations (12 and 24 months CA) computed for the communication precursors analyses is indicated in Table 2.

Scores of the 5 Griffiths subscales ate the age of 24 months Ca are reported in Table 3.

Interestingly, at 24 months CA, all subscales (Developmental quotient [DQ] in Locomotor, Personal Social, Eye and Hand coordination performance scales, and Global developmental quotient [GQ]) were within a normal range (+1 SD), whereas the mean Hearing and Speech Scale was within a normal range (but −1 SD), as shown in Table 4.

Table 4 shows the Pearson correlation coefficient between GMDS-R and antecedents of communication. The Pearson correlation coefficient displayed a direct correlation between the total number of utterances and the Hearing and Language subscale of the GMDS-R. Furthermore, a direct correlation was also found between eye contacts and the above mentioned GMDS-R subscale. Moreover, a highly significant and direct correlation was also present between the Personal-Social Scale of the GMDS-R and the Vocal Turns, the Non-Looking Vocal Turns, and the Total number of Utterances subscales. Finally, the GQ of the GMDS-R directly correlates to the Vocal Turns and the Total number of Utterances. The highest correlations were observed in the subscales assessing the more strictly communicative and linguistic aspects, in which greater interaction between adult and child was required.

## 4. Discussion

Early exploratory abilities are the base of linguistic skills [23]. The Video Analysis allows a direct evaluation during developmental follow-up visits. To our knowledge, the method presented here has not been used previously to monitor communication precursors in preterm infants with normal hearing and neurodevelopmental outcomes. Our protocol differs from Bortolini’s original assessment since it requires a shorter observation time. Furthermore, one video analysis can be reassessed without additional sessions.

Our data show that the development of the communicative intention progresses from a gestural communication to a verbal communication with an increase in both voice shifts and the number of statements.

ELBW at 12 months CA infants use predominantly eye contacts and gestural turns to communicate with adults. In contrast, at 24 months CA, the child’s communicative intention shifts from gestural communication, to verbal communication, characterized by an increase in both vocal turns and number of utterances produced. Moreover, this study revealed an increase in the number of utterances produced over time, a finding consistent with Caselli’s results [24]. According to this model, a child experiences a “word spurt”/“naming explosion” around 24 months of age, while gestural turns decrease, highlighting the passage from a gestural to a verbal communication mode.

Language development also depends on cognitive processes (e.g., memory, attention, etc.), [25]. In our populations, the performances at the GMDS-R scales are within a normal range. However, the average at the Hearing and Language sub-scale DQ is within −1 SD. Even though the cut-off for abnormality is 2 SD, it its noteworthy that nearly 70% of ELBW have a DQ below 1 SD [26]. Some investigators suggest that language difficulties in children born preterm depend on abnormal brain connectivity between brain regions, such as the cerebellum and corpus callosum [27].

In the current study, prerequisites related to communication development correlate positively with the GMDS-R Personal-Social and Hearing and Language subscales, which assess a child’s language and communication development. Assessment of development of the communication skills precursors across a preterm infant in the first two years of life is important as it allows to provide useful reference values for prediction of developmental delay. A few clinical interventions for promoting healthy language development exist currently [28]. Interventions that enhance preterm infants’ exposure to maternal speech are suggested as potential strategies for improving short-term health outcomes and also to reduce the risk of future delays in language development [28,29]. The results of this study suggest that the video recording technique is sensitive to change and could be used to assess the outcomes of early intervention.

However, it would be important to assess reference values as a predictive index of communicative, linguistic and neurodevelopment delay. A recent review reports a great individual variation in early vocalizations and babbling [15]. For this reason, routine follow-up visits could be enriched by a video analysis evaluation of communicative and language development in children at high risk of language impairment. Identification of predictors of long-term outcomes is also particularly relevant to help health care [30] in cases burdened by the impact of family socio-economic problems. It could give the possibility to implement customized intervention programs [31].

In addition, this technique could allow remote assessment of children’s functions during the pandemic era, or by different professionals belonging to facilities located in different centers.

An important limitation of this study is the lack of full-term infants as a control group. The lack of control group does not allow us to compare the precursors of communication with normal full-term neonates. Further studies are needed to standardize this video recording technique in order to validate the tool. The goal is to evaluate the precursors of communication and language development in children under 24 months of age with a validated tool. This would allow planning of early interventions in high-risk children.

Another limitation is the lack of reporting of other factors that could influence language development such as maternal education level or family context. As the first aim of this study was to evaluate language development in ELBW infants by the mean of the video analysis technique, the design was focused towards how child–adult interactions have been expressed.

## 5. Conclusions

ELBW infants at 12 months CA use predominantly eye contacts and gestural turns to communicate with adults. At 24 months CA, the child’s communicative intention evolves from gestural to verbal communication. Verbal communication is characterized by an increase in both vocal turns and the number of utterances produced during interaction. The total number of utterances and eye contacts were significantly correlated to the Development Quotient (DQ) of Hearing and Language scale. The video analysis we implement appears to be a sensitive tool for early assessment of communication and language development and to refine early intervention.

## Figures and Tables

**Table 1:** Means and standard deviations (SD) of number of communication precursors during video-recording at 12 and 24 months of CA are reported. Sample size = 45 cases.

Communication Precursors 12	12 Months CA (*n* = 45)		24 Months CA (*n* = 45)	
	Mean	SD	Mean	SD
Eye Contacts	171.38	78.49	229.36	76.46
Vocal Turns (VT) with eye contacts	1.74	1.05	4.67	2.67
Vocal Turns (VT) without eye contacts	1.10	0.82	2.76	2.13
Gestural Turns	5.31	1.76	3.36	1.56
Utterances	3.56	3.50	23.26	12.83

**Table 2:** Paired sample *t*-test between the two observations (12 and 24 months CA) computed for number of communication precursors in the video recording analysis. The statistical level of significance was *p* < 0.001 for all variables.

	Difference in Means	Std. Dev	Paired Difference 95% Confidence Interval of the Difference	
			Lower	Upper
Eye contacts	−57.97	74.55	−81.21	−34.74
Vocal turns	−2.92	2.20	−3.61	−2.23
Gestural turns	1.95	1.97	1.33	2.56
Non-looking vocal turns	−1.66	1.93	−2.26	−1.06
Utterances	−19.69	12.47	−23.737	−15.64

**Table 3:** Griffiths Mental Development Scale (GMDS-R). Means and standard deviations refer to the evaluation at 24 months CA (*n* = 45). Percentage of cases with neurodevelopmental impairment according to the Griffith Scales is reported.

	Means	Std. Dev	Neurodevelopmental Impairment (%)
Locomotor Scale	100.09	14.08	6.6%
Personal Social Scale	106.91	15.02	8.8%
Hearing and Language Scale	94.35	20.53	17.8%
Eye and Hand Coordination Scale	107.85	13.88	4.4%
Performance Scale	102.62	11.11	6.6%
General Quotient	102.26	14.92	8.8%

**Table 4:** Pearson correlation coefficients between GMDS-R version and video recording analyses. (**) = *p* < 0.005, (*) = *p* < 0.05.

	Locomotor	Personal Social	Hearing Language	Eye and Hand	Performance	General	Quotient
Eye contacts	Pearson CC	0.156	0.110	0.336	0.287	0.062	0.182
	Sig. (2-tails)	0.431	0.510	0.045 (*)	0.073	0.366	0.273
Vocal turns	Pearson CC	0.056	0.416	0.327	0.127	0.082	0.345
	Sig. (2-tails)	0.414	0.002 (**)	0.052	0.588	0.401	0.034 (*)
Gestual turns	Pearson CC	0.028	0.142	0.130	0.131	0.093	0.089
	Sig. (2-tails)	0.681	0.396	0.451	0.057	0.174	0.558
Non-looking							
vocal turns	Pearson CC	0.184	0.110	0.288	0.087	0.125	0.289
	Sig. (2-tails)	0.401	0.510	0.890	0.207	0.068	0.102
Utterances	Pearson CC	0.118	0.485	0.350	0.099	0.125	0.401
	Sig. (2-tails)	0.087	0.002 (**)	0.036 (*)	0.151	0.068	0.013 (*)

## Data Availability

The data presented in this study are available from the corresponding author on request due to privacy restrictions.
